# Cases and Observations Illustrative of the Connexion between Certain Forms of Pneumonia and Renal Disease

**Published:** 1856-07

**Authors:** Benjamin George M‘Dowel

**Affiliations:** Physician to the Whitworth and Hardwicke Hospitals, &c. &c.


### RECORD OF MEDICAL SCIENCE.

*Cases and Observations Illustrative of the Connexion between Certain Forms of Pneumonia and Renal Disease.* By BENJAMIN GEORGE M'DOWEL, A. B., M. D., Physician to the Whitworth and Hardwicke Hospitals, &c. &c.

The object of the following communication is to direct attention to a combination of morbid conditions, which, as far as I know, has not hitherto been *specially* noticed, but which, it is conceived, may prove of some use in extending our knowledge of the etiology of certain diseases. I now allude to the frequent coexistence of certain suppurative inflammations with chronic renal disease, but more especially to the combination of the latter with the suppurative and gangrenous forms of pneumonia, of which a considerable number of cases have come under my observation within a comparatively short period.

This combination of morbid conditions has occurred too frequently to allow me to regard it as a mere coincidence; and I think I shall be able to furnish good reasons for adopting the opinion:—1. That when pneumonia occurs in connexion with renal disease, it has a remarkable tendency to assume the suppurative or gangrenous form; and 2. That we are to regard the morbid condition of the kidney as the predisposing cause of these very fatal terminations of a disease which otherwise terminates favorably in the vast majority of instances.

At a recent meeting of the Pathological Society (January 19, 1856,) I exhibited a specimen of gangrenous pneumonia, in which a diseased condition of the kidneys likewise existed. The particulars of this case will be detailed further on (see Case IX.): but the observations then made, and which have a direct reference to the subject under consideration, were as follows:—" Dr. M'Dowel particularly directed the attention of the Society to this combination of disease, and stated his belief that chronic renal disease had a great influence on the development of suppurative inflammations. This he had especially observed in pneumonia, as, for the last eighteen months, during which period he had paid particular attention to this subject, *he had not met with a fatal case of suppurative pneumonia in which renal disease was not found to exist.* His opinion was, that pneumonia, in the great majority of instances, passes through certain regular phases, which naturally tend to a restoration of the healthy condition of the lung: but that when pneumonia occurred in a person in whom renal disease had pre-existed, the tendency of the disease then was to assume the suppurative, or the gangrenous form."

My object now is to adduce the evidence on which this opinion was founded.

*Double Pneumonia. Purulent Infiltration of the Lungs. Bright's disease of the Kidney. Death on the sixth day.*

Thomas Wood, a carman, thirty-two years of age, was admitted into the Hardwicke Hospital, under Dr. M'Dowel's caie, August 5, 1853. Patient was a man of very intemperate habits. Three days before admission (Sunday) he got drunk, and was exposed all night to wind and rain. He returned home early on Monday, feeling chilled and ill; violent rigors followed, with cough and oppression of the breathing.

On admission (fourth day, Wednesday), the face was darkly flushed, the dyspnœa intense, and there was total prostration cf strength. Respiration 44; pulse 130 in the minute; skin extremely hot; there was frequent cough, with thin, scanty, colorless expectoration; and pain on inspiration in the left side.

Double pneumonia was found to exist, the right lung being principally engaged; more than half of this lung gave the signs of solidification; over the portion of the left lung friction likewise existed, denoting the co-existence of pleuritis.

Fifth day. Right lung wholly solidified.

Respiration 52; pulse 140: raved incessantly all night; dyspnœa more urgent; vomiting; died on Thursday evening, sixth day of the disease.

*Autopsy. Thoracic Viscera.*—Double pneumonia, with pleuritis on the left side. The right lung was solidified throughout, and infiltrated with dark colored pus; the tissue of the lung was very friable and noncrepitant. The left lung was solidified in patches, in all of which pus was diffused. The uninflamed portion of the lung was emphysematous.

The pericardium was extremely adherent to the heart; in some places the adhesions consisted of bands of half an inch long. The heart was enlarged and hypertrophied, weighing 16 oz. when separated from the pericardium. Its tissue was slightly softer than natural, but its valvular apparatus was perfectly healthy.

*Abdominal Viscera.*—The liver was large and pale. The spleen softened, but of natural size.

*The Kidneys were extensively diseased;* externally both were pale, smooth, and mottled. The tubular structure had nearly altogether disappeared, and white, soft, fatty-looking material occupied the greater bulk of the organ. The left was much diminished in size The right weighed 5½ oz., and the left 2½ oz. only; in the latter a fibrinous clot of a brown color lay in a small cavity, with the walls of which it had but a very slight connection.—(*Pathological Register of the Whitworth and Hardwicke Hospitals*).

Six other cases of the co-existence of pneumonia with renal disease, are given; we omit them, as they are very similar to the above.

If we exclude Case V., which terminated in circumscribed pneumonic abscess, and in which life was prolonged for forty-seven days, the cases now detailed are all very similar. In them we see pneumonia running a very rapid course, terminating fatally in from six to nine days, and death the result of a profound impression on the system generally, rather than of asphyxia; for, as a general rule in these cases, dyspnœa was not even complained of; as if the nervous influence was so much lowered, or overpowered, that animal sensibility had become almost extinct. The condition of the kidneys was likewise very similar in all, being large, soft, and pale, and the seat of some adventitious deposit, which, more or less extensively, replaced the normal structure. In one case only was a different condition found to exist (Case IV.), and in it the kidney was sacculated or multilocular, and contained much pus. As it is usually met with, pneumonia is a disease which naturally tends to recovery, and which I can confidently state requires no heroic treatment to subdue it; but when it occurs in connection with an unsound condition of the kidneys (being *dependent*, it may be, on such condition), we are presented with a totally different form of disease, and one which too often will terminate fatally, notwithstanding the best directed treatment. And it may be deserving of future investigation to inquire, whether the danger which so notoriously attends "typhoid pneumonia," of which disease each one of the preceding cases, except Case IV., might be taken as a type, may not be found to depend upon the fact, that this form of disease is developed as the result of some constitutional fault, which, when traced to its source, may be found to originate in renal degeneration.

Direct evidence, I think, has now been furnished of the influence which renal disease exercises on the course, duration, and result of pneumonic inflammation, and especially of the tendency of pneumonia, under such circumstances, to pass into suppuration. On the other hand, if, in fatal cases of pneumonia, in which suppuration had not occurred, the kidneys were found healthy, we would have an additional argument in support of this opinion; but as pneumonia rarely proves fatal unless suppuration has first occurred, this evidence is more difficult to be procured; it is furnished, however, by the following case.

*Pleuro-pneumonia. Death from suffocative Catarrh on the thirteenth day. No pus in the lung. Kidneys healthy.*

John M'Kenna, aged 60, was admitted into the Hardwicke Hospital, under Dr. M'Dowel's care, June 5, 1851. The day before he was attacked with shiverings, headache, and pain in his left side, accompanied by such lassitude that, to use his own expression, "he was knocked down by it at once;" although he was so short a time ill, he was extremely weak. The chest was carefully examined, but only some bronchitis on the left side could be detected. Pulse 100, and compressible. He was ordered camphor mixture, with aromatic spirit of ammonia. His condition remained the same until the 8th, when he became much worse; he raved, and had become much weaker. Pulse 88, weak and intermittent. The lower half of the left lung was now found to be solidified; the lower half of that side of the chest was dull to percussion, and over the same extent the vascular murmur was replaced by tubular breathing. Bronchitis existed to a considerable extent on the right side; expectoration scanty, viscid and lemon-colored. To be dry-cupped extensively; a blister to be subsequently applied over the dull side; blue pill, carbonate of ammonia, and ipecacuanha in pill; wine 8 oz.

12th. Is much improved. Pulse 56, and regular; crepitus reduced, and diminution of bronchial breathing on the left side.

Continued steadily improving until the 15th, when it was found that alarming symptoms had become suddenly developed. His lips were livid; there were extreme dyspnœa, and a cold, damp, condition of the surface; effusion had taken place into the tubes, and a loud, mucous rattle was universally present. A large blister was applied between the shoulders, a mustard emetic administered, whilst wine was given liberally.

16th. No improvement; no emetic effect had been produced; whiskey and ammonia were given freely, but the patient gradually sank, and died the same night, being the thirteenth day of the disease.

*Autopsy.*—Right lung largely distended with air, and œdematous; much frothy serum exuding when incisions were made; bronchial tubes on both sides filled with a similar fluid; recent lymph effused on left pleural surfaces generally, but more especially in the region of the diaphragm. Lower lobe of the left lung completely hepatised; it sank in water, and was friable, but did not contain any pus. The heart was healthy; the kidneys were congested, but healthy.

The termination of pneumonia in gangrene may likewise be found associated with renal disease. In four instances, which have very recently come under my observation, this association of morbid conditions existed; but how far this combination of disease is to be regarded as the general rule, or its exception, must be determined by a more extended series of observations.

The author then details four cases of gangrenous pneumonia, in all of which disease of the kidneys existed.

These cases (12 in number) constitute the evidence in support of the opinion, already several times expressed, that between pneumonia in its worst and most fatal forms, and structural disease of the kidneys, some definite relationship exists. If this opinion be adopted, and if we regard the combination of diseases illustrated by these cases, as representing parts in one great chain of pathological sequences, then the order of these sequences is easily traced out. It is no new doctrine that a diseased condition of the kidneys cannot long exist without an impairment, more or less serious, of the great function of excretion. A morbid condition of the blood is established as the result of imperfect depuration; hence arise certain secondary affections, of which (in connection with diseased kidneys) serous inflammation and affections of the brain and heart have attracted the most attention. But it is probable that the secondary affections which depend on organic renal disease will vary according to the stage of the disease, or according to the peculiar form under which it is manifested.

"Bright's disease," we now know is a generic term which includes several dissimilar diseased conditions of the kidney; and, whilst inflammations of the serous membranes may be more frequently associated with one particular form of disease—viz., the small, contracted "granular" kidney—pneumonia in its worst forms (gangrenous or suppurative) may be more peculiarly the secondary results of the large, smooth, pale kidney, which was so uniformly found to coexist in the cases already detailed.

In these observations my object has been to avoid theorizing, and to furnish a simple statement of clinical facts; and I am perfectly ready to adopt or reject the opinions here advanced, as more extended observation may either confirm or refute them.

At another time I hope to be able to furnish some evidence of the influence which renal disease exercises on the development of pyemina in general; and, in fine, will very briefly state the conclusions which I think may naturally be deduced from the cases given in the preceding pages:—

1. That in fatal cases of pneumonia renal disease is very frequently found to exist.

2. That where such a combination of disease exists, suppuration of the lung will be very constantly met with.

3. That a similar morbid condition of the kidney is often found in gangrene of the lung.

4. Or, it may be conversely stated, that where pneumonia supervenes in a person, in whom renal disease has previously existed, it is very apt to assume the suppurative or the gangrenous form.

5. That pneumonia, when it occurs in such fatal forms, and under such circumstances, is probably one of the "secondary affections" of "Bright's disease."

It has not been my intention to convey the idea that pneumonia has never been enumerated by authors as one of the complications of "Bright's disease;" though some writers are silent on the subject, others have distinctly noticed it, especially Rayer. But it will be found that the pneumonia described in this paper is of a different nature from that noticed by Rayer. The pneumonia which he describes as secondary to "albuminous nephritis" comes on at a late period, and after the dropsy and other symptoms of the renal disease have been fully pronounced; and hence he observes:—"The symptoms of such pneumonias are more or less marked by those of dropsy, or by the symptoms of cardiac disease, or other concomitant pulmonary lesions." In none of the cases above described was renal disease suspected to exist; all were apparently in the enjoyment of good health up to the moment of the sudden development of the pulmonary affection. Neither do the cases of pneumonia, alluded to by Rayer and other authors (Bright, Gregory, Christison), resemble, in their anatomical characters, those detailed in the preceding paper. Suppuration and gangrene, either of which was found in all these instances, were not observed by any of these authors. One of Rayer's cases, however, is an exception to this statement—Case XXXVIII.—in which external suppuration of the lung was found found to exist. In the other two (for only three cases are given by Rayer in illustration) red hepatization and œdema, with engorgement of the lungs, were the principal morbid changes.

